# Intralymphatic Immunotherapy with Ultrasound Guidance Seems to Be Associated with Improved Clinical Effect in Canine Atopic Dermatitis—A Retrospective Study of 109 Cases

**DOI:** 10.3390/ani14202921

**Published:** 2024-10-11

**Authors:** Nina Maria Fischer, Claude Favrot, Franco Martini, Ana Rostaher

**Affiliations:** Dermatology Unit, Clinic for Small Animal Internal Medicine, Vetsuisse Faculty, University of Zurich, Winterthurerstrasse 260, 8057 Zurich, Switzerland; cfavrot@vetclinics.uzh.ch (C.F.); fmartini@vetclinics.uzh.ch (F.M.); arostaher@vetclinics.uzh.ch (A.R.)

**Keywords:** allergen-specific immunotherapy (AIT), intralymphatic immunotherapy, canine atopic dermatitis (CAD)

## Abstract

**Simple Summary:**

Intralymphatic immunotherapy is a treatment used for allergic diseases in both humans and animals. While ultrasound-guided injections were initially common, palpation-based injections have become more popular in veterinary dermatology. Precise injection into the lymph node seems crucial for the treatment’s effectiveness; therefore, this study aimed to evaluate the impact of injection method (ultrasound guidance vs. palpation-based) on clinical responses in dogs with atopic dermatitis. The retrospective analysis of 129 canine atopic dermatitis cases treated with ILIT between 2014 and 2022 found that dogs receiving ILIT via ultrasound guidance had a significantly higher success rate compared to palpation-based injections. These findings suggest that injection quality may influence treatment outcomes. Further research is needed to validate these findings.

**Abstract:**

**Background:** Intralymphatic immunotherapy (ILIT) has been used successfully in both human and veterinary medicine as a safe and effective treatment for allergic diseases. Initially, ILIT was administered by ultrasound guidance, but palpation-based injections have become more popular among veterinary dermatologists. Data from human medicine, however, show that precise injection into the lymph node is mandatory, and injection quality clearly correlates with clinical response. **Hypothesis:** Our aim was to assess the impact of the injection method (ultrasound guidance versus palpation-based guidance) on clinical response in dogs with atopic dermatitis. **Methods:** A total of 129 canine atopic dermatitis (CAD) cases treated with ILIT between 2014 and 2022 were retrieved from the hospital clinical database. The included dogs had to receive at least three intralymphatic injections administered either by palpation or ultrasound guidance. Those cases were retrospectively assessed and compared regarding clinical response to ILIT. **Results:** In total, 84 dogs received ILIT by ultrasound guidance, and in 25 dogs, ILIT was injected based on palpation. The success rate of ILIT was significantly higher in the ultrasound guidance group when compared to palpation-based injections. **Conclusions:** Low-quality injections must be considered as a possible reason for ILIT failure in dogs. Further prospective and controlled studies are necessary to confirm these results.

## 1. Introduction

Intralymphatic immunotherapy (ILIT) has been used in both human and veterinary medicine as a safe and effective intervention for allergic diseases [1,2,3,4,5]. Initially, ILIT was administered directly into the lymph node by ultrasound guidance, but it is the authors’ observation that palpation-based injections are becoming more popular with veterinary dermatologists [5]. A human study showed that precise injection into the lymph node correlated with positive clinical response [1]. This clearly questions the efficacy of palpation-based injections. Given that the allergen dosages employed in ILIT are significantly lower—approximately 100-fold—compared to subcutaneous immunotherapy (SCIT), the precision of intralymphatic injections may indeed hold paramount importance in eliciting both clinical and immunological responses [1].

The authors successfully performed ILIT and demonstrated that it leads to a faster and higher response rate compared to SCIT in canine atopic dermatitis (CAD) [6,7]. Data on administration techniques are missing in veterinary medicine, which led us to review our cases. The goal of this retrospective study was to assess the association between the ILIT injection technique and clinical response.

## 2. Materials and Methods

**Figure 1:**
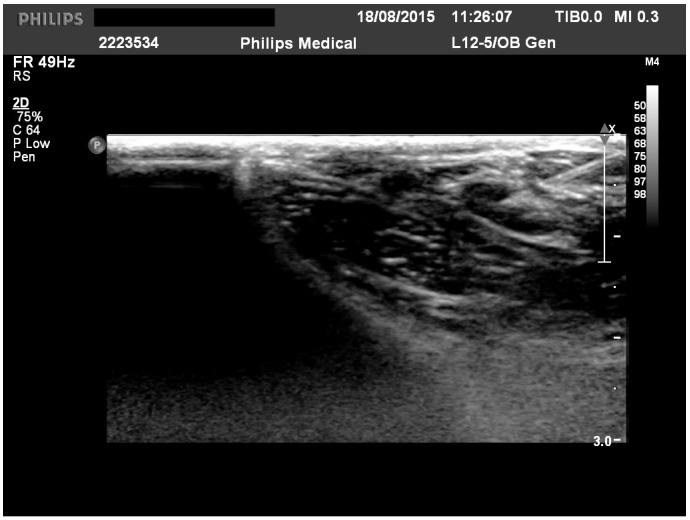
Illustration of an intralymphatic injection into the popliteal lymph node.

CAD cases treated by ILIT between 2014 and 2022 were retrospectively assessed regarding clinical outcome after 3 to 6 months of ILIT. Atopic dermatitis was diagnosed based on current criteria [8]. Dogs were included only when they had non-seasonal pruritus, and food allergies were excluded beforehand. Allergen solutions included aqueous allergens provided by a commercial company (Heska AG; Fribourg, Switzerland) and included up to 7 allergens (an average of 3) based on the sensitization pattern and clinical history of each dog. The allergen solution was mixed with aluminum hydroxide (Alhydrogel 2%, InvivoGen; San Diego, CA, USA) at a 1:1 ratio, and 0.2 mL was injected every 4 weeks as described previously [6]. Dogs not following this protocol were excluded from this study. Injections were performed at least 3 times and were either performed by ultrasound guidance (a board-certified radiologist, Figure 1) or by palpation (a board-certified dermatologist) into one popliteal lymph node. The efficacy of the intervention was assessed by comparing the recorded clinical presentation and the drug application at the ILIT start and at the last day of ILIT injections. The dog was clustered in the positive response group (responder dogs) when the symptomatic treatment (oclacitinib, topical or systemic glucocorticoids) could be reduced and, at the same time, the owner and investigator both evaluated the clinical presentation as improved (comparing the status at the start of ILIT and last day of ILIT injections).

Dogs receiving systemic antibacterial or antifungal therapy during the ILIT injection period were excluded from this study.

A statistical analysis was conducted to evaluate the influence of various variables on the success of intralymphatic immunotherapy (ILIT). The variables included the injection methodology, the weight of the dogs (classified as either less than 5 kg or greater than 5 kg), the number of injections administered (classified as either more than 4 injections or less than 4 injections), and the breed of the dogs. Statistical significance was determined using appropriate analytical methods to assess the potential impact of these factors on ILIT outcomes. As the assessed variables were categorical, Fisher’s exact test was used for the statistical analysis (GraphPad Prism version 9 Software, San Diego, CA, USA).

## 3. Results

In total, 129 CAD patients were treated with ILIT between 2014 and 2022, and 109 cases met the inclusion criteria.

**Figure 2:**
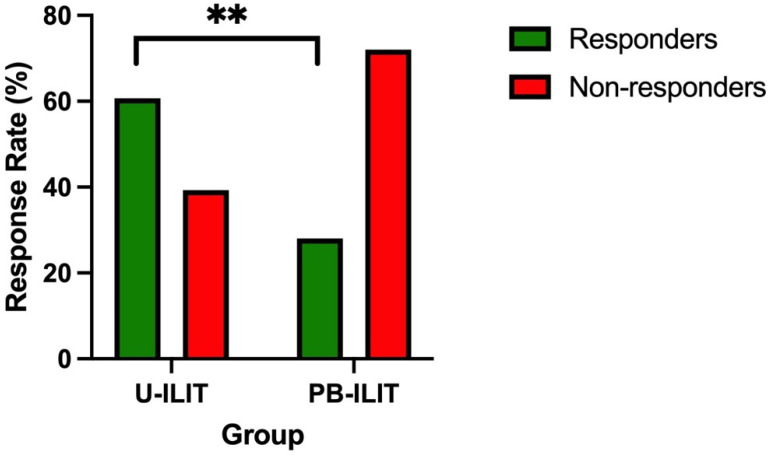
Response rate towards ILIT therapy (U-ILIT: intralymphatic injection by ultrasound guidance; PB-ILIT: palpation-based intralymphatic injection); ** significance (*p* = 0.005).

The study population comprised various dog breeds. The most frequently represented breeds were West Highland White Terriers (n = 9), French Bulldogs (n = 15), Labrador Retrievers (n = 7), Mixed Breeds (n = 12), English Bulldogs (n = 5), Boxers (n = 4), Jack Russell Terriers (n = 4), German Shepherds (n = 4), and Pugs (n = 4). The mean age of the dogs was 3.5 years, with individual ages ranging from 1 to 10 years. The gender distribution included 59 males and 50 females. For a comprehensive list of all breeds, ages, and genders, please refer to the Supplementary Data Table S1. In 84 dogs, ILIT was injected by ultrasound guidance (U-ILIT), whereas in 25 dogs, ILIT was injected palpation-based (PB-ILIT). In the U-ILIT group, 60.7% (51/84) responded positively compared to 28% (7/25) in the PB-ILIT group (Figure 2). Therefore, significantly more dogs responded to ILIT when it was injected by ultrasound guidance compared to palpation-based injections (*p* = 0.005).

The number of injections varied: 13 dogs received three, 67 dogs received four, 17 dogs received five, and 12 dogs received six injections. There was no statistically significant correlation between the response and number of injections, but a *p*-value of 0.08 suggests a trend that fewer injections showed a better outcome. As well as this, we showed that neither weight (*p* = 0,36) nor breed had a significant influence on ILIT response. For the breed-specific analysis, only breeds with more than 10 individuals were included, namely West Highland White Terriers (WHWTs; n = 11) and French Bulldogs (n = 18). *p*-values of 0.37 and 0.28, respectively, indicating that the response rates in these specific breeds do not differ significantly from those observed in the general population.

## 4. Discussion

This study presents novel findings that contribute to the understanding of intralymphatic immunotherapy (ILIT) in the management of CAD. The primary discovery is that the method of ILIT administration significantly affects clinical response, with ultrasound-guided injections (U-ILIT) yielding a higher success rate compared to palpation-based injections (PB-ILIT). This observation aligns with evidence from human medicine, which has demonstrated that precise injection into the lymph node is essential for positive clinical response [1,9].

Several limitations apply to this study. The retrospective nature of this study did not allow for the collection of objective clinical scores like CADESI [10] and PVAS [11]. As a result, the assessment of the ILIT response was based on the subjective evaluations of both the owner and the dermatologist, as well as data on the reduction in symptomatic treatments taken from the medical records. Despite this, the consistent assessment method across both groups mitigated potential bias (U-ILIT and PB-ILIT).

The unequal distribution of the patient number in both groups (84 versus 25 dogs) could have led to a better outcome in the U-ILIT group. However, this study did find a statistically significant difference in the response rate between the two groups (*p* = 0.005), indicating that the U-ILIT group had a higher success rate compared to the PB-ILIT group. This suggests that the difference in response rates is unlikely to be solely due to the uneven distribution of patients. The significance test used in this study (Fisher’s exact test) is appropriate for categorical data and can handle unequal sample sizes.

To ensure the robustness of the analysis, it would be ideal to conduct a prospective and controlled study to confirm these findings. Such a study could ensure a more balanced distribution of patients between groups, which would strengthen the conclusions drawn from the analysis.

Another potential limitation of this retrospective study is the short follow-up period after the final ILIT injection. As described in our study methodology, our analysis focused on the period immediately following the ILIT injections. However, it would be prudent to consider setting the endpoint at a later time, such as one month after the final ILIT injection or even one year after the first ILIT injection. This approach could help account for seasonal variations in the allergic conditions of the dogs. Although we selected non-seasonal atopic dogs, seasonal variations can still occur in these dogs, leading to improvements or the worsening of their condition based on the seasons.

Maybe performing palpation-based ILIT more often and gaining more experience could influence the results. A study by Müller et al. demonstrated that intralymphatic injections exhibit comparable efficacy to subcutaneous injections across different protocols. This finding suggests that experienced veterinarians can perform accurate palpation-based injections without the need for ultrasound guidance [5]. It should be noted that even injections performed by ultrasound guidance may be inaccurate, especially in non-cooperative dogs. Furthermore, from ultrasound imaging, the palpation of and injections made in the popliteal lymph nodes can be difficult due to their location in the popliteal space surrounded by subcutaneous fat and muscle tissue. These issues may clearly influence the ILIT outcome.

Unfortunately, cases with suboptimal injections have not been recorded herein, and consequently, the effect of this variable could not be studied. However, suboptimal injections were less likely in our population, as we did not see any correlation between breed and weight and ILIT outcome, and furthermore, the efficacy of the U-ILIT was in the expected range of previous studies (60%) [7].

## 5. Conclusions

In conclusion, this study seems to confirm the results from Skaarup et al. [1] that a positive clinical response seems to depend on successful injections and that these low-quality injections may be a possible reason for ILIT failure. Administration by ultrasound guidance and trained intralymphatic injection administration are, therefore, recommended. Furthermore, we would like to emphasize that this is a preliminary report arising from a retrospective collection of data. As such, further prospective and controlled studies are necessary to confirm these results and provide a more comprehensive understanding of the findings.

## Data Availability

Data are contained within the article and Supplementary Materials.

## Supplementary Materials

The following supporting information can be downloaded at: https://www.mdpi.com/article/10.3390/ani14202921/s1, Table S1: list of all breeds, ages, and genders of dogs.
