# Supplementary material for: Intralymphatic Immunotherapy with Ultrasound Guidance Seems to Be Associated with Improved Clinical Effect in Canine Atopic Dermatitis—A Retrospective Study of 109 Cases
Source: Animals (Basel). 2024 Oct 11;14(20):2921. doi: 10.3390/ani14202921 (PMC11503735; doi:10.3390/ani14202921)
Supplement: Supplementary file 1 [file animals-14-02921-s001.zip › animals-3127699-supplementary.pdf]

#	Outcome	breed	age	sex	weight	injections (#)	methodology	medication reduction	clinical improvement (subjectively assesed by owner & vet)
1	non-responder	WHWT	3	m	< 5kg	< 4	palpation-based	not possible	improvement
2	non-responder	Mix Breed	3	m	> 5kg	< 4	palpation-based	N/A	no improvement
3	non-responder	Rhodesian Ridgeback	4	f	> 5kg	< 4	palpation-based	not possible	no improvement
4	non-responder	Flat Coated Retriever	3	f	> 5kg	< 4	palpation-based	not possible	improvement
5	non-responder	WHWT	2	f	< 5kg	< 4	palpation-based	not possible	no improvement
6	non-responder	WHWT	5	f	< 5kg	< 4	palpation-based	N/A	no improvement
7	non-responder	Boxer	3	f	> 5kg	< 4	palpation-based	not possible	no improvement
8	non-responder	english Bulldog	8	f	> 5kg	< 4	palpation-based	not possible	no improvement
9	non-responder	Golden Retriever	2	m	> 5kg	> 4	palpation-based	not possible	no improvement
10	non-responder	Labrador Retriever	2	m	> 5kg	>4	palpation-based	not possible	improvement
11	non-responder	Pug	1	m	< 5kg	< 4	palpation-based	not possible	improvement
12	non-responder	Jack Russel terrier	6	m	< 5kg	> 4	palpation-based	N/A	no improvement
13	non-responder	WHWT	4	m	< 5kg	< 4	palpation-based	N/A	no improvement
14	non-responder	German Shepard	8	m	> 5kg	< 4	palpation-based	not possible	no improvement
15	non-responder	French Bulldog	3	m	< 5kg	< 4	palpation-based	not possible	no improvement
16	non-responder	Iceland hound	3	f	< 5kg	> 4	palpation-based	not possible	no improvement
17	non-responder	Cocker Spaniel	1	f	< 5kg	< 4	palpation-based	not possible	no improvement
18	non-responder	American Staffordshire Terrier	2	f	> 5kg	< 4	palpation-based	not possible	no improvement
19	responder	Boxer	1	m	> 5kg	< 4	palpation-based	N/A	improvement
20	responder	Magya Vizsla	7	m	> 5kg	< 4	palpation-based	possible	improvement
21	responder	Magya Vizsla	6	f	> 5kg	< 4	palpation-based	possible	improvement
22	responder	Basset Bleu de Gascogne	3	f	> 5kg	< 4	palpation-based	N/A	improvement
23	responder	Wolfspitz	4	f	> 5kg	< 4	palpation-based	possible	improvement
24	responder	French Bulldog	5	m	< 5kg	< 4	palpation-based	possible	improvement
25	responder	German Shepard	1	m	> 5kg	> 4	palpation-based	possible	improvement
1	non-responder	Golden Retriever	8	m	> 5kg	> 4	ultrasound-guided	not possible	no improvement
2	non-responder	WHWT	7	m	< 5kg	< 4	ultrasound-guided	not possible	no improvement
3	non-responder	Mix Breed	1	f	> 5kg	>4	ultrasound-guided	not possible	improvement
4	non-responder	French Bulldog	1	f	< 5kg	> 4	ultrasound-guided	not possible	no improvement
5	non-responder	Boxer	2	f	> 5kg	< 4	ultrasound-guided	not possible	no improvement
6	non-responder	French Bulldog	1	m	< 5kg	> 4	ultrasound-guided	not possible	improvement
7	non-responder	Labrador Retriever	3	m	> 5kg	> 4	ultrasound-guided	not possible	improvement
8	non-responder	Mix Breed	6	m	< 5kg	> 4	ultrasound-guided	not possible	no improvement
9	non-responder	Chihuahua	4	m	< 5kg	< 4	ultrasound-guided	not possible	improvement
10	non-responder	Mix Breed	8	f	< 5kg	< 4	ultrasound-guided	N/A	no improvement
11	non-responder	French Bulldog	3	f	< 5kg	< 4	ultrasound-guided	not possible	no improvement
12	non-responder	French Bulldog	2	f	< 5kg	< 4	ultrasound-guided	not possible	no improvement
13	non-responder	Sheltie	1	m	< 5kg	< 4	ultrasound-guided	not possible	no improvement
14	non-responder	english Bulldog	5	m	> 5kg	< 4	ultrasound-guided	not possible	no improvement
15	non-responder	Continental Bulldog	1	f	> 5kg	< 4	ultrasound-guided	not possible	no improvement
16	non-responder	French Bulldog	2	f	< 5kg	> 4	ultrasound-guided	not possible	no improvement
17	non-responder	Pudel	1	m	< 5kg	< 4	ultrasound-guided	N/A	no improvement
18	non-responder	Mix Breed	3	m	< 5kg	< 4	ultrasound-guided	not possible	no improvement
19	non-responder	French Bulldog	1	m	< 5kg	< 4	ultrasound-guided	not possible	improvement
20	non-responder	Mischli0g	7	m	< 5kg	< 4	ultrasound-guided	not possible	no improvement
21	non-responder	French Bulldog	3	m	< 5kg	< 4	ultrasound-guided	not possible	no improvement
22	non-responder	Poodle	4	m	< 5kg	< 4	ultrasound-guided	N/A	no improvement
23	non-responder	Biewer Yorkshire Terrier	4	m	< 5kg	< 4	ultrasound-guided	N/A	no improvement
24	non-responder	english Bulldog	9	f	> 5kg	> 4	ultrasound-guided	N/A	no improvement
25	non-responder	French Bulldog	1	f	< 5kg	< 4	ultrasound-guided	not possible	no improvement
26	non-responder	Mops	2	f	< 5kg	> 4	ultrasound-guided	not possible	no improvement
27	non-responder	Mops	5	m	< 5kg	< 4	ultrasound-guided	not possible	improvement
28	non-responder	Beagle	3	m	< 5kg	> 4	ultrasound-guided	not possible	no improvement
29	non-responder	Mix Breed	6	m	< 5kg	< 4	ultrasound-guided	not possible	no improvement
30	non-responder	Deutscher Wachtelhu0d	7	f	> 5kg	< 4	ultrasound-guided	not possible	no improvement
31	non-responder	Beagle	4	f	< 5kg	> 4	ultrasound-guided	not possible	no improvement
32	non-responder	Mops	1	m	< 5kg	< 4	ultrasound-guided	not possible	improvement
33	non-responder	WHWT	1	m	< 5kg	< 4	ultrasound-guided	not possible	no improvement
34	responder	WHWT	2	m	< 5kg	< 4	ultrasound-guided	possible	improvement
35	responder	Airdaile Terrier	9	m	> 5kg	> 4	ultrasound-guided	possible	improvement
36	responder	Golden Retriever	6	f	> 5kg	> 4	ultrasound-guided	N/A	improvement
37	responder	French Bulldog	3	f	< 5kg	< 4	ultrasound-guided	possible	improvement
38	responder	WHWT	2	m	< 5kg	< 4	ultrasound-guided	possible	improvement
39	responder	German Shepard	1	m	> 5kg	< 4	ultrasound-guided	possible	improvement
40	responder	Galgo Espagnol	5	m	> 5kg	< 4	ultrasound-guided	N/A	improvement
41	responder	English Bulldog	2	f	> 5kg	> 4	ultrasound-guided	possible	improvement
42	responder	French Bulldog	5	f	< 5kg	< 4	ultrasound-guided	possible	improvement
43	responder	Jack Russel Terrier	1	f	< 5kg	< 4	ultrasound-guided	possible	improvement
44	responder	Mix Breed	1	m	< 5kg	< 4	ultrasound-guided	possible	improvement
45	responder	Shar-Pei	2	f	> 5kg	> 4	ultrasound-guided	possible	improvement
46	responder	Beagle	3	f	< 5kg	< 4	ultrasound-guided	possible	improvement
47	responder	WHWT	3	m	< 5kg	< 4	ultrasound-guided	possible	improvement
48	responder	Mix Breed	4	m	> 5kg	> 4	ultrasound-guided	possible	improvement
49	responder	Sheltie	1	m	< 5kg	> 4	ultrasound-guided	N/A	improvement
50	responder	Boston Terrier	6	f	< 5kg	< 4	ultrasound-guided	possible	improvement
51	responder	Jack Russel Terrier	2	f	< 5kg	< 4	ultrasound-guided	possible	improvement
52	responder	Cockerpoo	8	f	< 5kg	< 4	ultrasound-guided	possible	improvement
53	responder	Mix breed	1	f	> 5kg	< 4	ultrasound-guided	N/A	improvement
54	responder	French Bulldog	4	f	< 5kg	> 4	ultrasound-guided	N/A	improvement
55	responder	Mini Bullterrier	3	f	< 5kg	< 4	ultrasound-guided	possible	improvement
56	responder	French Bulldog	6	m	< 5kg	< 4	ultrasound-guided	possible	improvement
57	responder	Lhasa Apso	1	m	< 5kg	> 4	ultrasound-guided	possible	improvement
58	responder	French Bulldog	2	m	< 5kg	< 4	ultrasound-guided	possible	improvement
59	responder	French Bulldog	3	m	< 5kg	< 4	ultrasound-guided	possible	improvement
60	responder	Mix Breed	6	f	> 5kg	> 4	ultrasound-guided	N/A	improvement
61	responder	German Shepard	2	f	> 5kg	< 4	ultrasound-guided	possible	improvement
62	responder	Shiba Inu	7	f	< 5kg	< 4	ultrasound-guided	possible	improvement
63	responder	Jack Russel Terrier	3	m	< 5kg	< 4	ultrasound-guided	possible	improvement
64	responder	Cairne Terrier	2	f	< 5kg	< 4	ultrasound-guided	possible	improvement
65	responder	Labrador Retriever	2	f	> 5kg	< 4	ultrasound-guided	possible	improvement
66	responder	Mix Breed	3	m	> 5kg	< 4	ultrasound-guided	possible	improvement
67	responder	Malteser	2	m	< 5kg	< 4	ultrasound-guided	possible	improvement
68	responder	French Bulldog	4	m	< 5kg	> 4	ultrasound-guided	possible	improvement
69	responder	Pug	2	m	< 5kg	> 4	ultrasound-guided	possible	improvement
70	responder	Mix Breed	1	f	> 5kg	< 4	ultrasound-guided	N/A	improvement
71	responder	Staffordshire Bullterrier	3	f	> 5kg	< 4	ultrasound-guided	possible	improvement
72	responder	French Bulldog	3	m	< 5kg	< 4	ultrasound-guided	possible	improvement
73	responder	Labrador Retriever	4	f	> 5kg	< 4	ultrasound-guided	possible	improvement
74	responder	Mix Breed	5	m	> 5kg	< 4	ultrasound-guided	possible	improvement
75	responder	WHWT	10	f	< 5kg	< 4	ultrasound-guided	possible	improvement
76	responder	Labradoodle	2	m	> 5kg	< 4	ultrasound-guided	possible	improvement
77	responder	WHWT	4	m	< 5kg	< 4	ultrasound-guided	N/A	improvement
78	responder	Shetland Sheepdog	3	m	> 5kg	< 4	ultrasound-guided	possible	improvement
79	responder	Deutscher Schäfer	2	m	> 5kg	< 4	ultrasound-guided	possible	improvement
80	responder	Chihuahua	3	f	< 5kg	< 4	ultrasound-guided	possible	improvement
81	responder	Continental Bulldog	4	f	> 5kg	< 4	ultrasound-guided	possible	improvement
82	responder	Labrador Retriever	3	f	> 5kg	< 4	ultrasound-guided	possible	improvement
83	responder	Labrador Retriever	3	m	> 5kg	>4	ultrasound-guided	N/A	improvement
84	responder	Labrador Retriever	5	m	> 5kg	< 4	ultrasound-guided	N/A	improvement
		WHWT: p=0.37, French Bulldog: p=0,28	mean= 3.5	m (n=58), f (n=50)	p=0,36	p=0,08			
